# Change in bronchial responsiveness and cough reflex sensitivity in patients with cough variant asthma: effect of inhaled corticosteroids

**DOI:** 10.1186/1745-9974-1-5

**Published:** 2005-08-25

**Authors:** Masaki Fujimura, Johsuke Hara, Shigeharu Myou

**Affiliations:** 1 Respiratory Medicine, Cellular Transplantation Biology, Kanazawa University Graduate School of Medicine, Kanazawa, Japan

## Abstract

**Background:**

Cough variant asthma (CVA) is a cause of chronic cough and a precursor of typical asthma. We retrospectively examined the longitudinal change in bronchial responsiveness and cough reflex sensitivity in CVA patients with respect to the effect of long-term inhaled corticosteroids (ICS).

**Methods:**

Provocative concentration of methacholine causing a 20% fall in forced expiratory volume in one second (PC20-FEV1) and provocative concentration of capsaicin eliciting 5 or more coughs (C5) were measured before treatment and during a follow up period following relief of cough (median; 2.0 (range; 0.5 to 8.0) years after the initial visit) in a total of 20 patients with CVA (7 males and 13 females, mean ± SD age of 49.9 ± 12.9 years).

**Results:**

Three of 8 patients not taking long-term ICS developed typical asthma compared to none of 12 patients taking ICS (p = 0.0171). PC20-FEV1 significantly (p < 0.0001) increased from 1.80 (GSEM, 1.35) to 10.7 (GSEM, 1.63) mg/ml in patients taking ICS but did not change in patients not taking ICS [2.10 (GSEM, 1.47) compared to 2.13 (GSEM, 1.52) mg/ml]. Cough threshold did not change in patients whether taking or not taking ICS.

**Conclusion:**

Long-term ICS reduces bronchial hyperresponsiveness in CVA as recognized in typical asthma. Cough reflex sensitivity is not involved in the mechanism of cough in CVA.

## Background

Cough variant asthma is a well-known cause of chronic non-productive cough as well as gastroesophageal reflux-associated cough and post-nasal drip-induced cough [1].

Pathophysiological features of cough variant asthma [2] appear to be similar to typical asthma, with mildly increased bronchial responsiveness and eosinophilic inflammation of central and peripheral airways, and a cough responsive to bronchodilator therapy [3]. It is, however, controversial whether cough reflex sensitivity contributes to the cough in CVA [4-7].

Johnson [8] reported that a significant proportion of patients diagnosed with cough variant asthma eventually develops wheezing, sometimes severe enough to require continuous bronchodilator therapy. Corrao et al. [3] reported that 2 of 6 patients with cough variant asthma began wheezing within 18 months of completing the study. Braman [9] restudied 16 patients diagnosed with cough variant asthma 3 to 5 years previously, and found that 37% of these patients manifested intermittent wheezing during the study period. Therefore, as nearly 30% of cough variant asthma patients have been demonstrated to develop typical asthma, cough variant asthma has been recognized as a precursor of typical asthma.

In our previous study [4], long-term inhaled corticosteroids (ICS) prevented the development of typical asthma from cough variant asthma. In another of our studies [5], longitudinal decline in pulmonary function in cough variant asthma was not different from that in healthy subjects and inhaled corticosteroids had no effect on the pulmonary function decline in cough variant asthma. However, it is unknown 1) whether bronchial responsiveness and cough reflex sensitivity change after relief of cough, 2) whether inhaled corticosteroids have an beneficial effect on bronchial responsiveness and cough reflex sensitivity, and 3) whether bronchial responsiveness increases after onset of typical asthma.

Although some researchers [6] reported that cough reflex sensitivity was increased in patients with cough variant asthma, our series of studies [4,5,7] have clearly demonstrated that cough reflex sensitivity is within normal limits in cough variant asthma as well as in stable typical asthma [10]. Cough reflex sensitivity is entirely independent of bronchial responsiveness [11] and bronchomotor tone [12]. Furthermore, cough reflex sensitivity does not change immediately after a patient's cough is completely relieved on therapy within 2 months [7]. Thus, abnormal cough reflex sensitivity is not considered to be essential in cough variant asthma.

We examined longitudinal changes in bronchial responsiveness and cough reflex sensitivity and influence of ICS on both responses in patients with cough variant asthma. Bronchial responsiveness to methacholine and cough reflex sensitivity to inhaled capsaicin were measured at least two times; at the initial visit and during the follow up period after relief of cough on treatment.

## Methods

Twenty patients with cough variant asthma as a single cause of chronic cough (median age 54 years, 7 men and 13 women), who had undertaken spirometry, bronchial reversibility test, methacholine provocation test, capsaicin cough provocation test, measurements of peripheral blood eosinophil count, serum total IgE and specific IgE to common allergens, and induced sputum eosinophil count at presentation, were followed up with special emphasis on typical asthma onset during 6 months or more (median 5 years, range 0.5 – 14) (Table 1). Spirometry and methacholine provocation test were repeated during the follow up period after their cough was completely relieved on the treatment.

**Table 1:** Clinical parameters in cough variant asthma patients with and without inhaled corticosteroids

	Without ICS	With ICS	P value	Total
Age (years)	53.3 ± 14.3	47.6 ± 12.0	0.3495	49.9 ± 12.9
Gender (male/female)	2/6	5/7	0.4439	7/13
Intreval of methcholine provocations (years)	2.7 ± 1.0	3.4 ± 2.8	0.5172	3.1 ± 2.2
	2.9 (1.1–4.0)*	2.0 (0.5–8.0)*		2.0 (0.5–8.0)*
Duration of illness (months)	41.5 ± 51.9	23.6 ± 31.4	0.3466	30.8 ± 40.5
	10.0 (2.0–120.0)*	12.5 (2.0–108.0)*		12.0 (2.0–120)*
Cough threshold (μM)	11.1 (1.63)**	6.2 (1.59)**	0.4163	7.8 (1.40)**
PC20-FEV1 (mg/ml)	2.13 (1.52)	1.80 (1.35)	0.7464	1.93 (1.27)
FVC (% predicted)	105.4 ± 14.3	103.1 ± 19.1	0.7765	104.0 ± 17.0
FEV1 (% predicted)	97.4 ± 15.2	93.2 ± 16.4	0.5763	94.9 ± 15.7
FEV1/FVC (%)	73.3 ± 6.9	78.1 ± 6.5	0.1318	76.2 ± 6.9

*; median (range), **; geometric mean (geometric standard error of the mean).

When the cough resolved on treatment with bronchodilators and/or inhaled and/or oral corticosteroids, we informed each patient that cough variant asthma is a precursor of typical asthma and induction of long-term inhaled corticosteroids (ICS) is desirable because the long-term therapy is recommended by many asthma guidelines in typical asthma even if the disease severity is mild. Long-term treatment with ICS was accepted and taken by 12 patients but not by the other 8 patients.

The diagnosis of cough variant asthma was made according to the following criteria proposed by Japanese Cough Research Society [13], excluding a criterion of cough reflex sensitivity within normal limits:

1) Isolated chronic non-productive cough lasting more than 8 weeks

2) Absence of a history of wheezing or dyspnea, and no adventitious lung sounds on physical examination

3) Absence of post-nasal drip to account for the cough

4) Forced expiratory volume in one second (FEV1), forced vital capacity (FVC), and FEV1/FVC ratio within normal limits (FEV1 ≥80% of predicted value, FVC ≥80% of predicted value, and FEV1/FVC ratio ≥70%)

5) Presence of bronchial hyperresponsiveness (provocative concentration of methacholine causing a 20% fall in FEV1 (PC20-FEV1) <10 mg/mL)

6) Relief of cough with bronchodilator therapy

7) No abnormal findings indicative of cough aetiology on chest roentgenogram

All patients with cough variant asthma had been successfully treated with bronchodilators and/or corticosteroids, without use of other medications such as proton pump inhibitors and histamine H1-antagonists. Thus, cough variant asthma was the single cause of chronic cough in all patients studied.

The efficacy of bronchodilator therapy described above was assessed according to the following criteria:

1) "Excellent" when cough totally resolved

2) "Good" when sleep and daytime quality of life were improved

3) "Fairly good" when severity and frequency of cough were somewhat decreased

4) "Poor" when cough was unchanged

An assessment of "Excellent" or "Good" was judged as effective.

Pulmonary function, cough reflex sensitivity, and bronchial responsiveness were measured in that order within two weeks of the first visit (Table 1), and then repeated during the follow up period after each patient's cough was completely relieved by the treatment. FVC, FEV1 and flow-volume curves were measured using a dry wedge spirometer (Chestac 11, Chest Co., Ltd., Tokyo, Japan). Spirometry was performed and evaluated according to the ATS criteria [14]. Capsaicin cough threshold (C5), a concentration of capsaicin solution eliciting 5 or more coughs, was measured as an index of cough reflex sensitivity [7,10-12]. A provocative concentration of methacholine causing a 20% or greater fall in FEV1 from prechallenge values (PC20-FEV1) was measured as an index of non-specific bronchial responsiveness [15].

The onset of typical asthma was defined as wheezing and/or dyspnoeic attack responding to bronchodilator therapy.

### Data analysis

Data excluding PC20-FEV1 and C5 were presented as mean ± standard deviation (SD). PC20-FEV1 and C5 were expressed as geometric mean value with geometric standard error of the mean. Differences between groups were determined by parametric one-way analysis of variance (ANOVA) or the χ^2 ^test. Changes within group were assessed using the paired t test. PC20-FEV1 and C5 were analyzed using logarithmically transformed values. A p value of 0.05 or less was considered significant.

## Results

**Figure 1:**
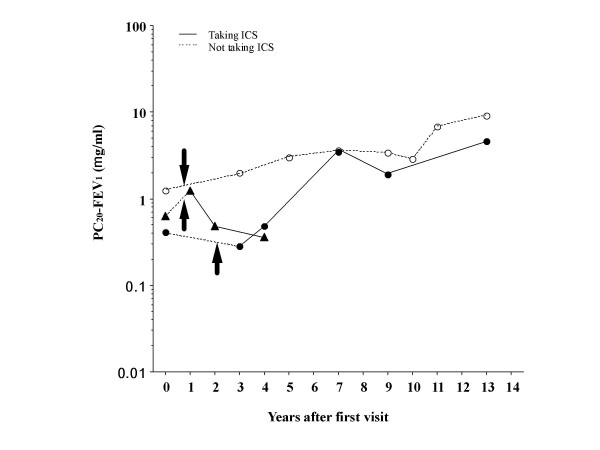
Longitudinal change in bronchial responsiveness in 3 patients with cough variant asthma who developed typical asthma while did not taking inhaled corticosteroids. PC20-FEV1, provocative concentration of methacholine causing a 20% or greater fall in forced expiratory volume in 1 second (FEV1), was determined by a mouth tidal breathing method. Bronchial responsiveness was not obviously increased following onset of typical asthma. ICS, inhaled corticosteroids. Arrows indicate onset of typical asthma.

Typical asthma onset was recognized in 3 (37.5%) of 8 patients not taking long-term inhaled corticosteroids (ICS) and none of 12 patients taking ICS. The prevalence of asthma onset was significantly (p = 0.0214) different between the groups. Details of the 3 patients who developed typical asthma are shown in Fig. 1.

Clinical parameters at initial presentation are summarized in Table 1. Median interval between the first and the second measurement of bronchial responsiveness was 2.9 years (range 1.1–4.0, mean (SD) 2.7 (1.0)) in the 8 patients not taking long-term ICS and 2.0 years (range 0.5–8.0, mean (SD) 3.4 (2.8)) in the 12 patients taking long-term ICS. The follow-up period was not significantly different between the groups. Age, gender, duration from onset of cough to presentation, capsaicin cough threshold, PC20-FEV1, FVC, FEV1 and FEV1/FVC ratio were not different between the two groups.

**Figure 2:**
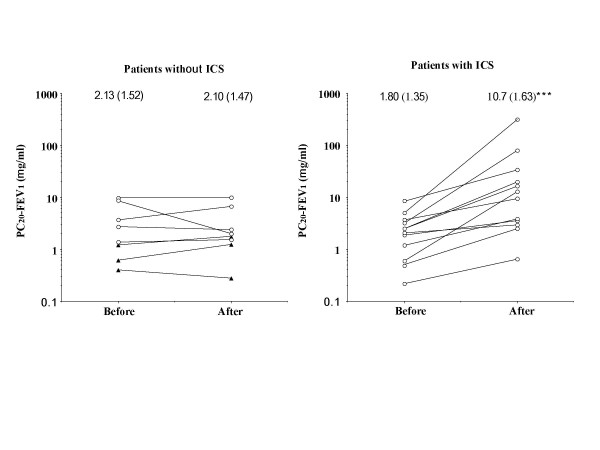
Longitudinal change in bronchial responsiveness in patients with cough variant asthma taking or not taking long-term inhaled corticosteroids. Closed triangles indicate patients developing typical asthma. ***p < 0.0001.

**Figure 3:**
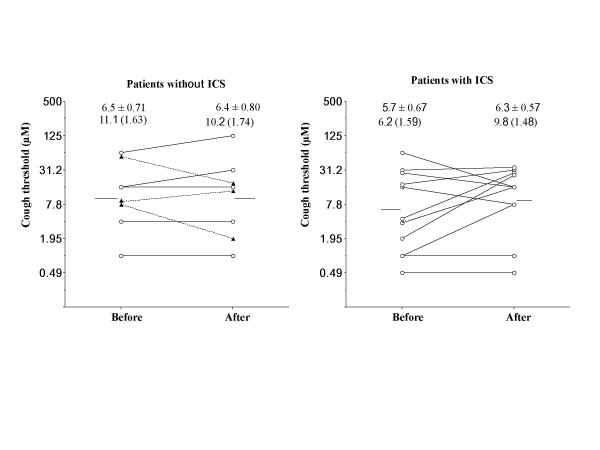
Longitudinal change in cough reflex sensitivity in patients with cough variant asthma taking or not taking long-term inhaled corticosteroids. Closed triangles indicate patients developing typical asthma.

**Figure 4:**
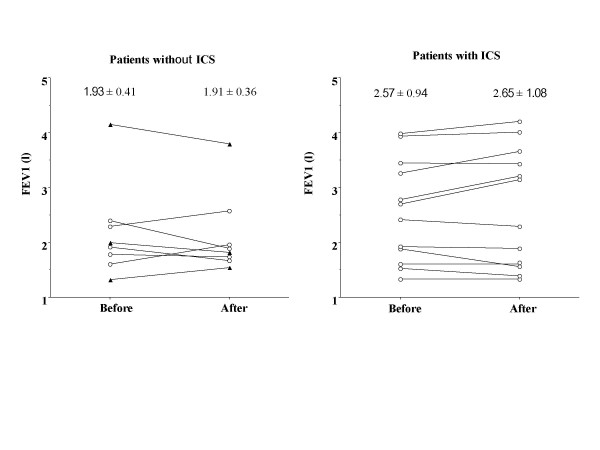
Longitudinal change in forced expiratory volume in one second (FEV1) in patients with cough variant asthma taking or not taking long-term inhaled corticosteroids. Closed triangles indicate patients developing typical asthma.

PC20-FEV1 significantly (p < 0.0001) increased by 5.9 (GSEM, 1.40) times from 1.80 (GSEM, 1.35) to 10.7 (GSEM, 1.63) mg/ml in patients taking ICS, but did not change in patients not taking ICS [by 0.97 (GSEM, 1.17) times from 2.13 (GSEM, 1.52) to 2.10 (GSEM, 1.47) mg/ml] (Fig. 2). PC20-FEV1 did not significantly change in the 3 patients who developed typical asthma [from 0.68 (GSEM, 1.38) to 0.89 (GSEM, 1.81) mg/ml] (Fig. 1, Fig. 2) or in 5 patients who did not develop typical asthma while they were not taking ICS [from 4.23 (GSEM, 1.46) to 3.52 (GSEM, 1.42) mg/ml] (Fig. 2). Capsaicin cough threshold (Fig. 3) or FEV1 (Fig. 4) did not change in patients whether taking or not taking ICS.

**Figure 5:**
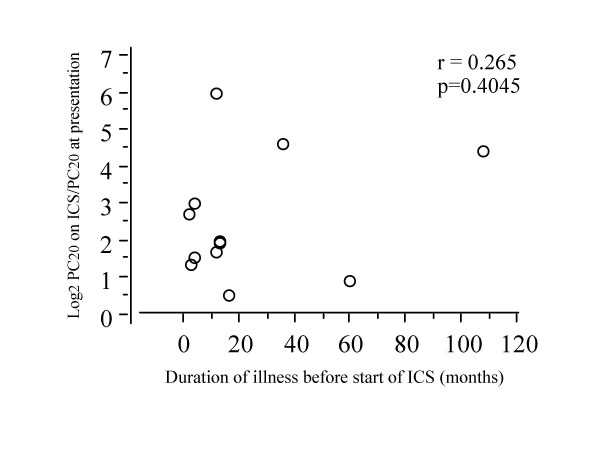
Relationship between duration of illness before induction of inhaled corticosteroids and degree of improvement of bronchial hyperresponsiveness in patients with cough variant asthma taking long-term inhaled corticosteroids.

**Figure 6:**
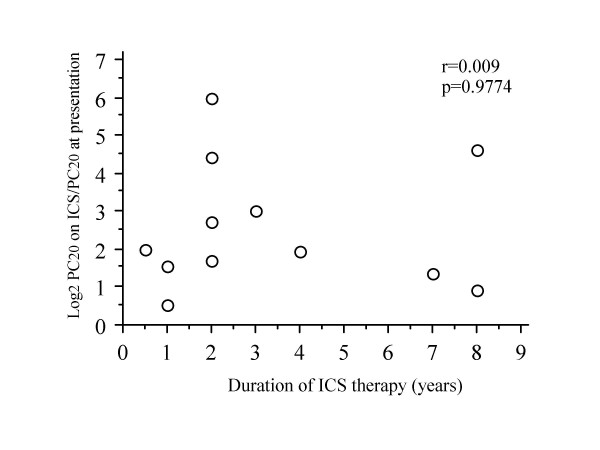
Relationship between duration of inhaled corticosteroid treatment and degree of improvement of bronchial hyperresponsiveness in patients with cough variant asthma taking long-term inhaled corticosteroids.

Change in PC20-FEV1 by long-term treatment with ICS did not correlate with duration from the onset of cough to the induction of ICS treatment (r = 0.265, P = 0.4045) (Fig. 5) or duration of ICS treatment (r = 0.009, p = 0.9774) (Fig. 6).

## Discussion

Cough variant asthma was first described by Glauser [16]. The only presenting symptom is isolated chronic cough responsive to bronchodilator therapy. The cough can occur for many years as an extremely annoying symptom interfering with work, sleep, and quality of life. Recognition of cough variant asthma is clinically important because bronchodilator therapy is an effective antitussive in cough variant asthma. Bronchodilators usually exert no antitussive effect in other causes of isolated chronic cough [17] such as post-nasal drip-induced cough, gastroesophageal reflux-associated cough [17], and atopic cough [4,5].

Nearly 30% of cough variant asthma patients eventually develop wheezing, sometimes severe enough to require continuous treatment with bronchodilators [3-5]. In this study, wheezing was recognized in none of 12 patients taking long-term inhaled corticosteroid (ICS) therapy and in 3 of 8 patients without ICS therapy. This result confirms our previous investigation [4] that the typical asthma onset rate was significantly lower in patients receiving ICS therapy, suggesting the utility of long-term ICS as an intervention against typical asthma onset from cough variant asthma.

The present study clearly showed that bronchial responsiveness did not change after relief of cough without use of ICS, and long-tem ICS attenuated bronchial responsiveness to inhaled methacholine in patients with cough variant asthma, probably resulting in prevention of development of typical asthma from cough variant asthma. There were only 3 patients developing typical asthma whose bronchial responsiveness was more increased among the patients not taking ICS and was not obviously increased after the asthma onset as shown in Fig. 1. These findings suggest that an increased bronchial responsiveness at presentation may be a risk factor for asthma development from cough variant asthma whereas further increase in bronchial responsiveness may not be necessary for the asthma onset. It is unclear why only coughing occurs and additional wheezing appears without change in bronchial hyperresponsiveness in this eosinophilic airway disorder.

It has been shown that early induction of ICS within 2 years following asthma onset is beneficial to achieve both control of symptom and improvement of pulmonary function and bronchial responsiveness in asthma [18]. Although number of patients taking long-term ICS was small in this study, there was no significant influence of duration of illness before induction of ICS on the degree of improvement of bronchial responsiveness. Niimi et al [19] have shown that airway remodelling exists but the extent is smaller in cough variant asthma than in typical asthma. This is likely to be responsible for the lack of influence of ill duration on effect of ICS on bronchial responsiveness. Further studies are needed to clarify this issue.

Although other researchers have reported that cough reflex sensitivity was heightened and recovered to a normal level following successful treatments of cough variant asthma [20-23], it should be recognized that cough reflex sensitivity is entirely independent of bronchial responsiveness [11] or bronchomotor tone [12], and that it is within normal limits in stable typical asthma [10]. We previously showed that 14 of 64 non-asthmatic healthy subjects (21.9%) had bronchial hyperresponsiveness when PC20-FEV1 of 10 mg/ml or less was defined as bronchial hyperresponsiveness [24]. In another of our studies [11], a C5 of 1.95 μM or less, 3.9 μM or less, and 7.8 μM or less was seen in 4 (5.6%), 14 (19.7%), and 31 (43.7%) of 71 non-asthmatic healthy subjects, respectively. Considering the proportion of subjects with bronchial hyperresponsiveness, it is considered that a C5 of 3.9 μM or less to be defined as cough reflex hypersensitivity. Thus, in the present study, cough reflex sensitivity was judged to be increased at initial presentation in 2 of 8 patients (25%) not taking ICS and 6 of 12 patients (50%) receiving ICS. These findings are not consistent with our previous findings that cough reflex sensitivity was within normal limits in cough variant asthma [4,5,7,10]. Nevertheless cough reflex sensitivity did not change after relief of cough despite use of ICS in the present study, confirming our previous findings that cough reflex sensitivity did not change following successful treatment of cough variant asthma [7]. Taken together, it can be concluded that cough reflex sensitivity is not involved in the mechanism of cough in cough variant asthma even when it is increased. In other words increased cough reflex sensitivity is not a primary feature of cough variant asthma and ICS does not affect the sensitivity. We do not know why eosinophilic airway inflammation does increase cough reflex sensitivity in atopic cough but not in cough variant asthma. Precise interaction between eosinophilic airway inflammation and cough reflex sensitivity should be disclosed by future studies.

Early induction of ICS within 2 years following asthma onset has been shown to be beneficial in attenuating bronchial hyperresponsiveness as well as achieving both control of symptom and improvement of pulmonary function [18]. In this study, the degree of reduction of bronchial hyperresponsiveness with ICS did not correlate with the duration between onset of cough and induction of ICS. It is not consist with the above-mentioned result on asthma [18]. A possible explanation of this discrepancy may be that airway remodelling increasing bronchial responsiveness such as subepithelial fibrosis and smooth muscle hypertrophy does not develop or become more severe as the duration of illness is longer, while thickening of subepithelial layer has been demonstrated in cough variant asthma [19]. This possibility needs to be clarified in future studies.

## Conclusion

The present retrospective study showed that bronchial hyperresponsiveness and cough reflex sensitivity did not change after relief of cough when ICS therapy was not taken in patients with cough variant asthma. A median of 2 years ICS treatment attenuated bronchial hyperresponsiveness, but not cough reflex sensitivity. Bronchial responsiveness did not further increase after onset of typical asthma in 3 patients not taking ICS. These findings suggest that long-term ICS treatment may prevent onset of typical asthma from cough variant asthma by reducing bronchial hyperresponsiveness, and that cough reflex sensitivity is not involved in mechanism of cough in cough variant asthma. Further studies including randomized placebo-controlled studies are needed to confirm the preventive effect of long-term ICS on typical asthma onset from cough variant asthma.

## List of abbreviations

ANOVA = analysis of variance, C5 = provocative concentration of capsaicin eliciting 5 or more coughs, CVA = cough variant asthma, FEV1 = forced expiratory volume in one second, FVC = forced vital capacity, GSEM = geometric standard error of the mean, ICS = inhaled corticosteroids, PC20-FEV1 = provocative concentration of methacholine causing a 20% fall in forced expiratory volume in one second, SD = standard deviation,.
